# SoloTE for improved analysis of transposable elements in single-cell RNA-Seq data using locus-specific expression

**DOI:** 10.1038/s42003-022-04020-5

**Published:** 2022-10-06

**Authors:** Rocío Rodríguez-Quiroz, Braulio Valdebenito-Maturana

**Affiliations:** Department of Genetics and Genomic Sciences, Icahn School of Medicine at Mount Sinai, New York, New York, USA

**Keywords:** Software, Gene regulatory networks

## Abstract

Transposable Elements (TEs) contribute to the repetitive fraction in almost every eukaryotic genome known to date, and their transcriptional activation can influence the expression of neighboring genes in healthy and disease states. Single cell RNA-Seq (scRNA-Seq) is a technical advance that allows the study of gene expression on a cell-by-cell basis. Although a current computational approach is available for the single cell analysis of TE expression, it omits their genomic location. Here we show SoloTE, a pipeline that outperforms the previous approach in terms of computational resources and by allowing the inclusion of locus-specific TE activity in scRNA-Seq expression matrixes. We then apply SoloTE to several datasets to reveal the repertoire of TEs that become transcriptionally active in different cell groups, and based on their genomic location, we predict their potential impact on gene expression. As our tool takes as input the resulting files from standard scRNA-Seq processing pipelines, we expect it to be widely adopted in single cell studies to help researchers discover patterns of cellular diversity associated with TE expression.

## Abstract (web summary)

SoloTE includes the genome location of transposable elements (TE) in scRNA-Seq data to determine how TE activity regulates neighboring gene expression.

## Introduction

Transposable elements (TEs) are molecular agents with the capability to move (“transpose”) within and between genomes^1^, and they are present in most eukaryotic genomes. TEs are organized into two classes: retrotransposons and DNA. Retrotransposons are subdivided into Long Terminal Repeats (LTRs), LINEs (Long Interspersed Nuclear Elements), and SINEs (Short Interspersed Nuclear Elements). Both LTRs and LINEs code for proteins involved in the retrotranscription of their mRNAs and subsequent insertion of these novel copies in another genomic location, whereas SINEs take advantage of the LINE proteins to retrotranspose^2^. On the other hand, DNA TEs code for a transposase, which excises the element from its original position, and inserts it elsewhere^2^. According to their sequence similarity, TEs from each class are further organized into families. Because of the potential negative impact of the transposition of TEs, most of them have accumulated mutations that render them inactive (also known as “Old TEs”), with few copies remaining intact (also known as “Young TEs”)^3,4^.

Although originally thought to be junk DNA, TEs are now being recognized as drivers of evolution^1^ and regulators of gene expression^5^. Their role in gene regulation is dependent on their genomic location, with some TEs having effects on neighboring genes (“cis effects”) or on genes located far away in the genome (“trans effects”)^6^. Thus, understanding TE expression in a locus-specific manner is paramount to assess their effect on modulating gene activity.

Bulk RNA-sequencing (RNA-Seq) is the gold standard method to profile gene expression across several types of tissues and/or across several experimental conditions^7,8^. However, because the source material is homogenized before sequencing, it captures an average portrait of gene expression, losing patterns of gene activity specific to certain cellular groups^4,8^. A recent advance of this technology is single-cell RNA-Seq (scRNA-Seq), which is now being widely adopted, because it allows to analyze gene expression across all cell types of a tissue^8^. scRNA-Seq has been successfully applied to profile tissues in healthy conditions (development^9^, regeneration^10^) and in diseases (cancer^11^, amyotrophic lateral sclerosis^12^, Alzheimer’s disease (AD)^13^), revealing novel patterns of gene expression and cell heterogeneity at an unprecedented scale.

TEs are often discarded in RNA-Seq analysis due to their repetitive nature, and this has severely hindered our understanding of their role in gene regulation. For bulk RNA-Seq, several tools have been developed to analyze locus-specific TE expression (reviewed by Lanciano & Cristofari^3^), however, similar developments for novel RNA-Seq techniques are scarce^4,14^. To the best of our knowledge, the only tool developed for the analysis of TEs in scRNA-Seq datasets is scTE^14^, which summarizes TE expression on metagenes corresponding to TE families, thus, losing the genomic location from which each TE is expressed. In turn, this limits the potential avenues that could be explored by analyzing TE activity. In this work, we present SoloTE, a tool to analyze TEs in scRNA-Seq data using locus-specific expression. First, we compare it to scTE, in terms of (i) detecting TE expression, (ii) UMAP dimensional reduction, and (iii) detection of cell marker TEs. Then, we applied SoloTE to three datasets. First, we studied the murine embryonic two-cell stage, in which TE expression is known to occur in a subset of cells^14^, and then in datasets whose TE expression has not been explored before: early gastric cancer (GC)^11^ and APP/PS1 AD mouse model^13^.

## Results

### Comparison with scTE

**Fig. 1:**
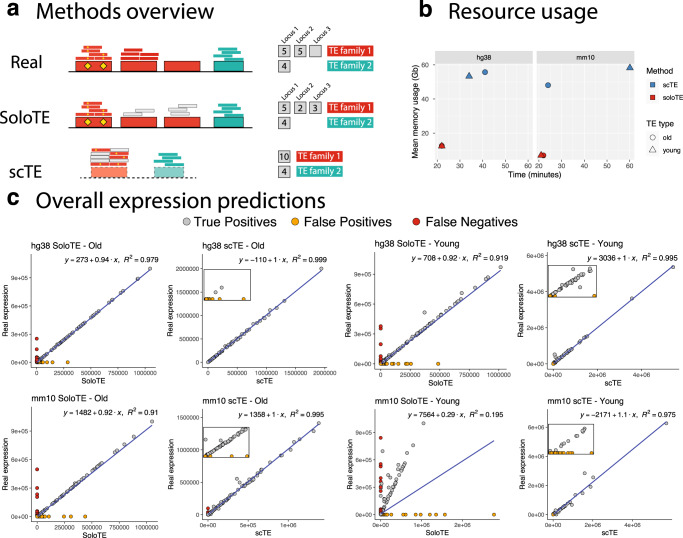
General comparison between SoloTE and scTE. **a** Overview of the read assignment per TE of each method. Four TEs are depicted, with three of them belonging to “family 1” (in red) and the remaining one to “family 2” (in cyan). Yellow diamonds indicate sequence variations specific to a TE. The smaller rectangles above each TE correspond to reads assigned to that specific location, with small gray rectangles corresponding to multi-mapped reads. The counts to the right indicate how each method calculates TE expression. “Real”: the expected situation in which 2 TEs from family 1 are expressed, and 1 TE from family 2 is expressed. “SoloTE”: reads are assigned to each locus, with multi-mapped reads assigned to a random location. “scTE”: dashed rectangles indicate that reads are assigned to TE metagenes, and summarized by families. **b** Memory usage versus time plots of SoloTE and scTE using the datasets simulated in this work: left, simulated data from the human genome hg38 version; right, simulated data from the mouse genome mm10 version. Color indicates the method (scTE in blue, SoloTE in red), and the shape indicates the TE type (circle for Old TEs, and triangle for Young TEs). **c** Overall expression estimates (without cell demultiplexing) for each simulated dataset. First row corresponds to the comparisons using the hg38 datasets, and the second row to the comparisons using the mm10 datasets. In each plot, the *x* axis corresponds to the method used to estimate expression (SoloTE or scTE), and the *y* axis to the real TE expression. Points are colored according to whether they match an expressed TE (gray, “True Positive”), TE mistakenly reported as expressed (orange, “False Positive”), TE expressed but not detected by the method (red, “False Negative”). Inset plots were added to the scTE main plots to show the spread of false positive predictions.

To the best of our knowledge, scTE is the only computational effort to include TEs in scRNA-Seq analysis, so we compared the performance of our tool against it. The main methodological difference between SoloTE and scTE, is that the former attempts to estimate the locus-specific expression of TEs, whereas the latter assigns read to TE metagenes summarized at the family level (Fig. 1a). We speculate that the approach used by scTE might under- or over-estimate TE expression, because of interfamily similarities that lead to read assignment ambiguity^3,4^. Indeed, when studying the different modalities of scTE, we found preliminary evidence to support this statement (Supplementary Table 1). In consequence, this could affect scRNA-seq analysis of specific cell types. Thus, we compared the tools first in general terms: resource (memory and time) usage and global estimation of TE expression (before cell demultiplexing); and in terms of scRNA-Seq specific metrics: UMAP dimensional reduction, and cell marker TE detection.

Regarding time and memory usage, scTE takes between 20 and 60 mins per run, whereas SoloTE takes <25 mins per run. Moreover, the memory usage of SoloTE was at most 15 Gb, while scTE used between 40 and 60 Gb per run (Fig. 1b). It is worth noting that before processing BAM files, scTE requires the generation of a genomic index, which can take between 5-42 minutes (Supplementary Table 2), in contrast to SoloTE that can directly process the BAM file. Altogether, we found that SoloTE outperforms scTE in computational resource usage. Then, to gain an idea on the overall TE expression levels estimated by each tool before cell demultiplexing, we compared them against the real TE expression. At this point, the expression across each cell was summed, and then used for the analysis (Fig. 1c, Table 1). TEs mistakenly reported as expressed by a tool were labeled as false positives (Fig. 1c, orange circles and Table 1), whereas TEs not detected were labeled as false negatives (Fig. 1c, red circles and Table 1). We found that for both hg38 and mm10 old TEs, the results obtained using each tool correlated well with the real expression levels, although SoloTE seems to have a few false negative results. In contrast, we observed that for young TEs there was a higher proportion of false positives and false negatives in the SoloTE results, since those TEs are the most problematic to assess in terms of genomic locations. As scTE reports result at the family level, and does not report TEs with locus resolution, comparing its output against our simulated ground truth data in terms of TE locus, is unfeasible. Thus, it is worth noting at this point, that the scTE results seem to have a smaller proportion of false positives, because we compared them against the expected results also aggregated at the family level. Collectively, these results indicate that both tools are able to detect TE expression, with a small proportion of wrongly assigned TEs. To better understand the estimations reported by each tool in a scRNA-Seq context-specific manner, we then assessed the impact in cell type clustering and analysis steps.

**Table 1:** Overall expression predictions.

Genome	TE group	Method	TP	FP	FN
hg38	Old	scTE	95.502	4.498	0
hg38	Young	scTE	81.9	18.1	0
mm10	Old	scTE	93.96	4.314	1.726
mm10	Young	scTE	78.723	21.198	0.079
hg38	Old	SoloTE	93.834	4.143	2.023
hg38	Young	SoloTE	55.959	39.806	4.235
mm10	Old	SoloTE	84.991	8.379	6.63
mm10	Young	SoloTE	26.06	64.162	9.777

For each simulated dataset, defined by the genome used (“Genome”) and the TE group (“Old” or “Young”), the performance of TE detection by “scTE” and “SoloTE” (“Method” column) is reported. “True Positives” (TP) correspond to TEs reported by the method that also are in the real expression matrix (i.e., a TE correctly identified), “False Positives” (FP) correspond to TEs reported by the method that are not actually expressed, and “False Negatives” (FN) correspond to TEs in the real expression matrix that were not detected by a method.

**Fig. 2:**
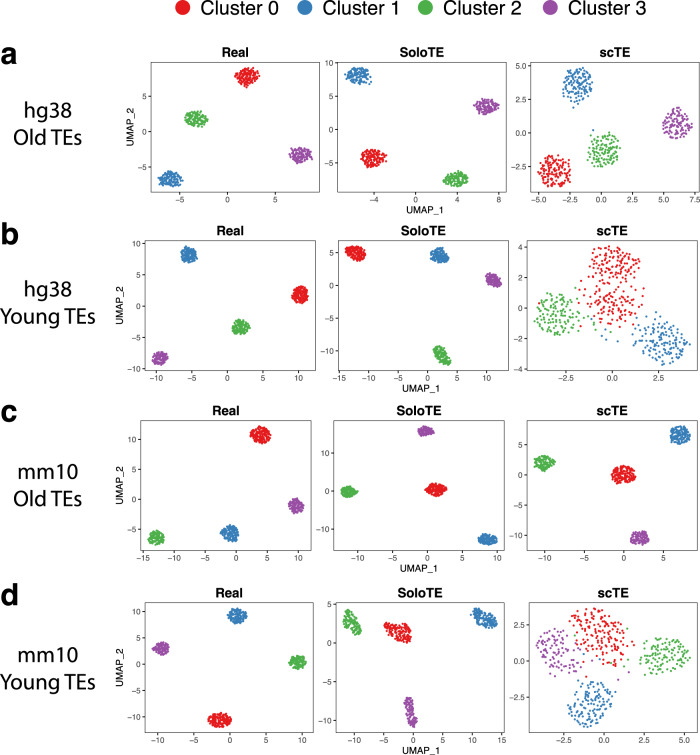
UMAP dimensional reduction plots for each of the simulated datasets. **a** hg38 Old TEs. **b** hg38 Young TEs. **c** mm10 Old TEs. **d** mm10 Young TEs. Above each UMAP plot, the dataset from which it was generated, is indicated: ground truth expression matrix (“Real”), SoloTE expression matrix (“SoloTE”), and scTE expression matrix (“scTE”). Clusters are colored red (Cluster 0), blue (Cluster 1), green (Cluster 2), purple (Cluster 3).

The gene-cell expression matrixes from the simulated experiment, along with those generated with SoloTE and scTE, were processed with Seurat (see Methods), in order to identify cell clusters and their corresponding TE markers. For old TEs in both hg38 and mm10, we found that through the use of either tool, the four expected clusters could be obtained (Fig. 2, “hg38 Old TEs” and “mm10 Old TEs”). Nonetheless, in neither case the cluster positions across the UMAP were the same as those in the real UMAP, probably as a consequence of the small error rates described in the previous paragraph. It is worth noting that through the use of the Seurat integration protocol, almost all TEs from the SoloTE matrix, could be associated with the corresponding expected cluster, while this could not be achieved when using the scTE matrix (Supplementary Figure 1). A different result could be seen for young TEs (Fig. 2, “hg38 Young TEs” and “mm10 Young TEs”). scTE clustering results have the cells more separated between them, while the clusters are closer, and even mixed. This is probably due to the indiscriminate assignment of TE expression to family metagenes, impeding the estimation of more subtle differences. This is further supported by the fact that SoloTE clustering results for young TEs reveal the 4 expected clusters.

**Fig. 3:**
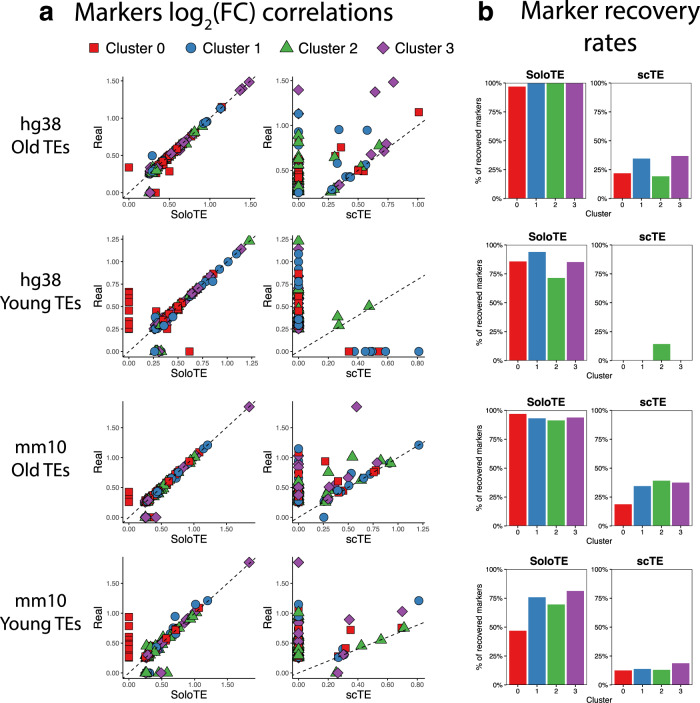
Comparison of predicted cell markers between SoloTE and scTE. **a** For each simulated dataset, correlation plots of the average log_2_(FC) are depicted, with the real value on the *y* axis, and the method (SoloTE or scTE) on the *x* axis. Cluster 0 markers are shown in red squares, Cluster 1 markers in blue circles, Cluster 2 markers in green triangles and Cluster 3 markers in purple diamonds. Dashed line at *y* = *x* was added to aid in visualization of under- and over-estimation of values. **b** Bar plots showing the rate of markers retrieved by each method. Cluster identifier is indicated on the *x* axis, whereas the percentage of correctly recovered markers on the *y* axis.

As one of the main focus in scRNA-Seq is to identify markers for each cell cluster, our final comparison between scTE and SoloTE was in terms of how well the expected TE cell markers could be retrieved when using the TE expression calculated by each tool. Across all datasets, we performed cell marker identification with Seurat (see Methods). We labeled a marker as correctly recovered, if there was a match with the expected result in terms of the TE identifier and the cluster to which it belonged, without imposing any log_2_(FC) threshold. In general, we found that SoloTE has a consistently high marker recovery rate, with the exception being the mm10 young TEs (Fig. 3). Particularly, our results indicate that these markers correlate well with the real markers. On the other hand, we found that in all cases, scTE severely underestimated log_2_(FC) values (points above dashed lines in Fig. 3). Moreover, scTE had a limited marker recovery rate, with one case being particularly low: a small number of markers of only 1 cluster were found, and none for the remaining 3 clusters (Fig. 3, “hg38 Young TEs”).

Collectively, these results depict that SoloTE represents an improvement over scTE, by taking advantage of locus-specific TE information. Based on these findings, we then aimed to uncover the TE repertoire across 3 datasets. First, on the murine embryonic two-cell stage, which was also used in the original scTE publication, because it represents an example of well-known TE expression in a specific cell population. Then, on early GC and in the APP/PS1 AD mouse model, both datasets in which TE expression at the single cell level has not been explored before.

### TE expression in the murine embryonic two-cell stage

**Fig. 4:**
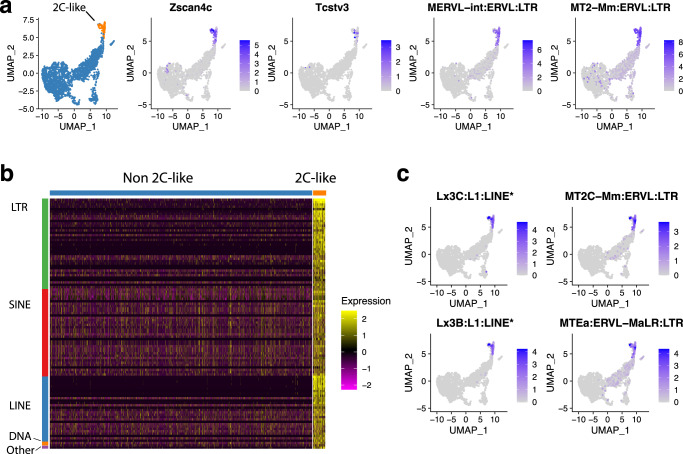
SoloTE analysis results during the murine embryonic 2-cell stage. **a** UMAP plots depicting cell groups (2C-like cells in orange, and non 2C-like in blue), 2C-like known cell markers (“Zscan4c” and “Tcstv3”), and previously known TEs expressed in 2C-like cells (“MERVL-int:ERVL:LTR” and “MT2-Mm:ERVL:LTR”). **b** Heatmap of all TEs differentially expressed in the 2C-like cells found using SoloTE. Cell types are indicated above (non 2C-like in blue, and 2C-like in orange), and TE classes are indicated on the left (LTRs in green, SINEs in red, LINEs in blue, DNA TEs in orange, and Other TEs in purple). **c** UMAP plots of TEs detected exclusively with SoloTE in the 2C-like cells. “*” denotes locus-specific TEs.

The embryonic two-cell stage is a key step in mouse development. Particularly, is characterized by zygotic genome activation, and it marks the shift from the use of the maternal genetic program toward the embryonic genetic program^15^. Current evidence shows that TEs contribute to the regulation of pluripotency during this stage^16^. Using the *Zscan4c* and *Tcstv3* known markers for the 2-cell (2C) like cell population, we then explored how TEs were expressed. First, we were able to confirm the expression of the MERVL-int:ERVL:LTR and MT2-Mm:ERVL:LTR, which are also known to be differentially expressed in the 2C-like cells (Fig. 4a). Using a log_2_(Fold Change) ≥1 threshold, we were able to find that there were no TEs expressed in the non-2C-like cells, and we identified a total of 106 marker TEs in the 2C-like cells (Fig. 4b). With our tool, we detected a greater number of marker TEs, when compared to the 28 markers reported by scTE (Supplementary Figure 2, Supplementary Data 1). These TEs spanned all major classes, with 38 (35.85%) LTRs, 37 (34.9%) SINEs, 28 (26.42%) LINEs, 2 (1.89%) DNA, and 1 (0.94%) Other TE. Some TEs exclusively identified with our tool are depicted in Fig. 4c, and correspond to the MT2C and MTEa LTR TEs and to locus-specific instances of the Lx3B and Lx3C LINE TEs. These TEs exhibit a similar pattern of cell expression as the known genes and marker TEs previously indicated. Collectively, the results obtained with this analysis confirm that SoloTE can identify known TEs across cellular groups, and add to the repertoire of expressed TEs those detected with locus resolution.

Locus-specific TE analysis revealed that 18 of them could be statistically associated with 12 genes (Methods). We found 5 genes having positive correlations with TEs, and 7 had negative correlations. Of these genes, only 7 (2 positive, 5 negative) could be associated with a biological process in the Panther classification system (Supplementary Data 2). The 2 positively-correlated genes found are *Map4* (Microtubule-associated protein 4) and *Tcf7l1* (Transcription factor 7-like 1). *Map4* is associated with neuron projection development and microtubule cytoskeleton organization, whereas *Tcf7l1* is associated with the Wnt signaling pathway and transcription regulation. On the other hand, the genes correlating negatively with TEs were *Gm9008* (Predicted pseudogene 9008), *Mapt* (Microtubule-associated protein tau), *Marveld2* (MARVEL domain-containing protein 2), *Scml2* (Scm polycomb group protein-like 2) and *Tfeb* (Transcription factor EB). Of these, *Marveld2* and *Tfeb* are associated with transcriptional regulation, whereas *Gm9008* is associated with protein ubiquitination, a process that has been linked to transcription factors important during cell reprogramming and associated with the 2C-like state^17,18^. The *Scml2* gene is involved in chromatin binding and *Mapt* with neuron projection development and microtubule cytoskeleton organization, similar to *Map4* mentioned earlier. In sum, this locus-specific analysis suggests that TEs could be modulating the expression of several genes involved in transcriptional regulation and other genes involved in microtubule-related processes, which are key for cell differentiation^19^.

### TE expression in early GC

TE expression has been reported to occur in several types of cancer^20–23^. Although most of these findings were obtained using bulk RNA-Seq data, they still point towards a trend in TE activity during the cancer disease state. Recently, scRNA-Seq has been used to profile several types of cancer, but to the best of our knowledge, the activity of TEs has not been explored. In this work, we focused our efforts in GC. In worldwide terms, this cancer represents the second cause of mortality^24^, following lung cancer^25^. Currently, the 5-year survival rate for early GC (EGC) is >90%, whereas for advanced GC is ~30%^26^. Because of this, there is a high interest in finding alternatives for detecting this cancer in its early stages.

**Fig. 5:**
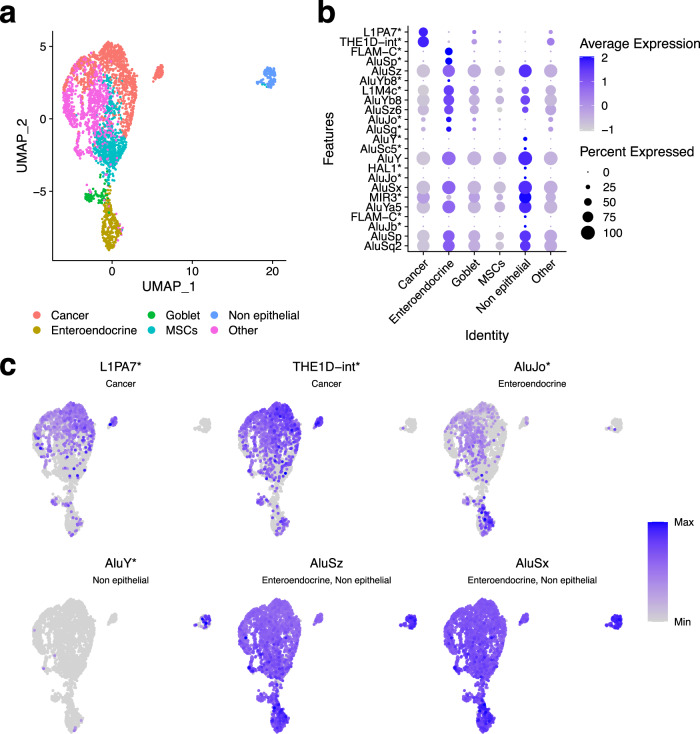
TE expression detected with SoloTE during early gastric cancer. **a** UMAP plot indicating the different cell types. **b** Dot plot depicting marker TEs per cell types (indicated on the *x* axis). **c** UMAP plots of selected TEs. Label below the TE identifier indicates the cluster at which the TE is a marker. First two TEs are differentially expressed in the cancer cells, second two TEs in the enteroendocrine and non-epithelial cells, respectively, and the last two TEs are expressed across all cells, but show increased expression in the enteroendocrine and non-epithelial cells. “*” denotes locus-specific TEs.

TEs have the potential to be used as markers, given that specific TEs can become active under certain conditions^27^. For example, they have been used as markers of aging^28^, and of lung cancer^29^. Taking this into account, we applied SoloTE to dissect the impact of TE expression in the EGC cellular heterogeneity. After processing with Seurat, we obtained a total of 6 cell clusters: Cancer, Enteroendocrine, Goblet, Metaplastic stem-like cells (MSCs), Non-epithelial, and other gastric cells (“Other”) (Fig. 5a). Then, we performed the marker analysis, and found a total of 24 TEs (compared to the three TEs obtained with scTE, Supplementary Figure 2 and Supplementary Figure 3). Moreover, we were able to find 2 TEs with locus resolution, having higher expression in the Cancer cell cluster when compared to the other cell types identified: L1PA7 (located at chrY:18989629-18990627) and THE1D (located at chr8:61579930-61580458) (Fig. 5b, c). Interestingly, we were able to also find TEs differentially expressed in enteroendocrine cells and non-epithelial cells (Fig. 5b, c). This finding suggests that TE activity in the early-malignant lesion in other cell types could be indicative of a global alteration in gene expression programs, which in turn, might contribute to the GC progression.

Following our statistical modeling approach to investigate the potential impact of the locus-specific expression of TEs (Methods), we found seven TEs associated with six genes (Supplementary Data 2). Amongst the genes having a positive correlation with TEs, we identified (function according to the Panther enrichment analysis in parenthesis): *SLC27A4* (secondary carrier transporter), and *MALAT1* (no results). On the other hand, we identified the following genes having negative correlations with TEs: *CDH1* (cadherin), *PAM* (oxygenase), *RUNX3* (Runt transcription factor), and *LINC-PINT* (no results). Overall, these results suggest that TEs could be potentially influencing transcriptional regulation (associations with *RUNX3*) and several cellular processes involved with transport and cell communication.

Of particular relevance to the disease, is that previous works have linked all of the aforementioned genes with cancer. Downregulation of *CDH1* and *LINC-PINT* has been implicated in GC^30,31^. The *PAM* and *RUNX3* genes have anti-cancer and tumor suppressor activities, and thus, their loss-of-function is characteristic of gastric and several other types of cancer^32,33^. Conversely, upregulation of *MALAT1*^34,35^ has been implicated in GC, whereas *SLC27A4* has also been reported to be overexpressed, but in other cancer types^36,37^.

Interestingly, the genes whose down-regulation is associated with cancer are those that we found to be negatively correlated with TEs. As we only analyzed marker TEs, a negative correlation would mean that for a given cell cluster, the TE has increased expression whereas the gene has decreased expression. In other words, this could suggest that the TE might be negatively regulating the gene. In turn, this could implicate TEs in contributing towards the disease phenotype. On the other hand, all of the genes reported to be up-regulated in cancer, positively correlate with TE expression. Based on the correlation analysis, it is not possible to discern whether the TE could be driving the increase in gene expression, or vice versa. Taken altogether, our locus-specific analysis underlines a putative role of TEs in GC, in agreement with the vision that TE expression in cancer can have several consequences on gene expression^38^.

### TE expression in APP/PS1 AD mouse model

**Fig. 6:**
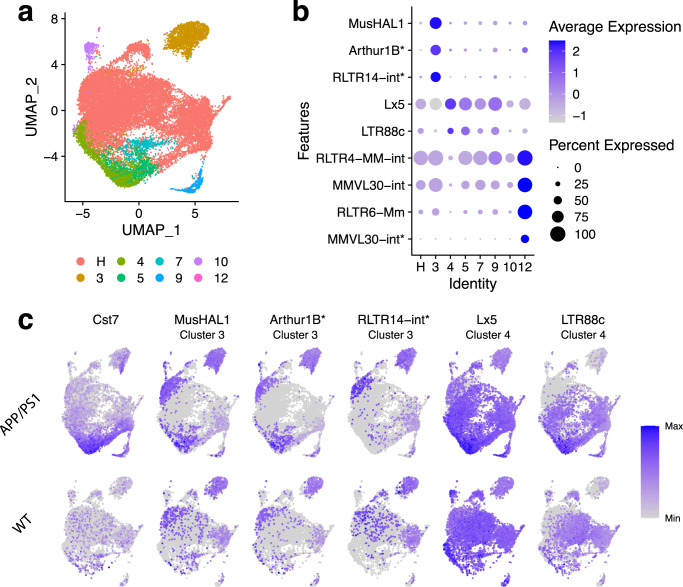
TE expression detected with SoloTE in the APP/PS1 Alzheimer’s disease mouse model. **a** UMAP plot indicating the different cell types (“H”: homeostatic cells). **b** Dot plot depicting marker TEs per cell types (indicated on the *x* axis), having a disease-specific effect. **c** UMAP plots of *Cst7* (known gene with increased expression in the disease) and selected TEs. Upper row corresponds to the expression in the AD APP/PS1 samples, whereas the lower row corresponds to the expression in the wild-type (WT) samples. Label below the TE identifier indicates the cluster at which the TE has increased expression. “*” denotes locus-specific TEs.

The last aim of our work was to investigate whether TEs are expressed during AD. Generally speaking, several previous works have reported de-repression and subsequent activation of TEs in neurodegenerative diseases, such as amyotrophic lateral sclerosis^4,39–41^ and AD^42–44^. Using a recently published dataset from the transgenic AD mouse model that carries the human APP/PS1 mutant genes, and their respective control wild-type samples^13^, we applied SoloTE to uncover the repertoire of TEs that are transcriptionally up-regulated in the disease samples. In the original work, according to the expression of marker genes, they merged several clusters into the Homeostatic (H) cluster, while the remaining clusters were associated with specific genes (Fig. 6a). Particularly, the authors annotated disease-associated microglia (DAM) clusters based on the expression of *Cst7*, *Lpl,* and *Clec7a*. We replicated this approach when processing the SoloTE cell expression matrix. In our analysis, clusters 4 and 5 correspond to the DAM group. After this, we performed a marker analysis with Seurat (see Methods) to detect TEs having increased expression in a given cell cluster when compared to the remaining ones. At this point, we obtained 71 markers TEs versus 0 obtained with scTE (Supplementary Figure 2).

To uncover which of the marker TEs have a disease-specific effect, we performed a Differential Expression (DE) analysis. To this end, we adopted a pseudobulk approach in which expression of genes and TEs per each cell cluster was aggregated, and then, edgeR was used to statistically test for differences (method reviewed by Squair et al.^45^). As a result of this strategy, we found a total of 9 TEs that are markers of cell clusters, and have increased expression in the disease samples when compared to wild-type (Fig. 6b, c). Of these TEs, 3 were detected at the locus-level: Arthur1B (marker of cluster 3, located at chr14:105919204-105919261), RLTR14-int (marker of cluster 3, located at chr7:55428645-55428856), and MMVL30-int (marker of cluster 12, located at chr2:85850484-85851460) (Fig. 6b).

Locus-specific analysis of these TEs, following the same approach for the datasets described earlier, revealed 1 TE correlating negatively with the *Olfr1033* (Olfactory receptor 1033) gene, and 1 TE correlating positively with the *Siglech* (Sialic acid binding Ig-like lectin H) gene (Supplementary Data 2). Panther analysis revealed that *Olfr1033* is a transmembrane signal receptor, whereas *Siglech* is involved with cell adhesion. Notably, olfactory dysfunction has been associated with AD^46,47^, indicating that the negative correlation we found between the MMVL30-int TE and *Olfr1033* could be of importance to understand the disease pathogenesis. Increased expression of several genes of the *Siglec* family has been associated with neurodegenerative diseases, and in particular, *Siglech* has been linked with amyotrophic lateral sclerosis^48^. Based on our analysis, it could be speculated that a TE-driven increase of *Siglech* expression could be contributing to the AD progression in the APP/PS1 mouse model.

Interestingly, we were able to find 2 marker TEs in the DAM clusters (Lx5, LTR88C), but not at the locus-level, impeding us to further analyze and predict their potential impact in AD. Similar to the result obtained for the early GC dataset, we observed that TE expression is spread to other cell types besides those associated with the disease (Fig. 6b, c). This discovery suggests that if TE expression is a driver of the aberrant phenotypes, it does so by having a direct influence (activation in disease-associated cell types) and by also having an indirect influence (activation in other cell types). Likewise, TE activation in other cell types could be a catalyst in the transition toward a diseased cell. Conversely, an alternative hypothesis to this, is that TE activation could be playing a regulatory role in the normal activity of cells, as has been reported to occur for several healthy states^1,4,9,10,49,50^.

## Discussion

TEs, which are often overlooked in gene expression analysis, have begun to gain recognition in several research groups. This could be attributed to the fact that there is increasing evidence associating them with development and other healthy states, along with pathologies such as cancer and neurodegenerative diseases. On par with the advancement of bulk RNA-Seq, several tools have been developed for the analysis of TE expression, with increased focus on accurately assessing the locus of activity. However, only one tool for single cell TE analysis from high-throughput scRNA-Seq data has been published, and it omits the location of TEs. As TEs can influence genes in their genomic vicinity, it is of importance to assess the expression without losing their positions. CELLO-Seq, a recent development, can measure TE expression at the locus-specific level with single-cell resolution. To achieve this, CELLO-Seq integrates long-read sequencing with a bespoke computational framework. This strategy revealed improvements in the mapping of both young and old TEs, yet for some “very young TEs” they were unable to map reads to specific genomic locations^51^. However, the methodology has only been shown to work with low number of cells (6 in mouse and 96 in human). On the other hand, SoloTE can be readily integrated and applied to data already generated from high-throughput scRNA-Seq technologies, which can profile between hundreds and thousands of cells^52–54^.

In this work, we presented SoloTE, an improvement over the previous tool for analysis of TEs in scRNA-Seq data. We found that our tool is faster and uses less computational resources than scTE. This is probably due to the fact that scTE requires the building and subsequent use of “TE indexes”, whereas SoloTE can start directly from the BAM file obtained with either CellRanger or STAR. In terms of usability, we found that scTE does not filter repetitive elements in such indexes, and thus, in the resulting matrix other types of non-TE repeats, such as Satellites, appear. Moreover, the identification of TEs to a non-TE expert could seem obscure, as no special keyword appear to differentiate TEs from genes in the expression matrix. Conversely, SoloTE adds the “SoloTE” keyword at the start of each TE id, making it more straightforward to identify and select TEs in any downstream analysis.

In terms of locus-specific TE analysis, SoloTE reports the location of expression if the reads could be accurately assigned to it. If not possible, it adopts the approach of summarizing TE expression at the family level. Considering that in most organisms a high proportion of the TE repertoire has accumulated mutations, most of them are sufficiently distinct from other copies, ensuring the unique alignment of reads. Nonetheless, a limitation of our tool is that for organisms with a greater proportion of transpositionally-active TEs, this could not be the case, as most of the copies could be genetically intact. Other limitations that could also impact TE expression measurements may be associated with the scRNA-Seq technology used. For 3’ end 10X single-cell data, a commonly used single-cell platform^55^, such limitations are: (i) not all TEs can be uniquely mappable with the short 100 nucleotides reads generated (Supplementary Figure 4 and Supplementary Figure 5), and (ii) events in which the TE could be acting as an alternative transcription start site cannot be detected due to the 3’ bias. Further improvements to our pipeline are the integration of the Expectation–Maximization (EM) algorithm and analysis of TE-derived isoforms. The EM algorithm reallocates reads to a TE locus based on the uniquely mapped reads (“expected” reads, E-step) surrounding that particular genomic location, and then through multiple iterations, assigns multi-mapped reads (M-step). Although this procedure has been a key element of bulk RNA-Seq TE analysis tools, such as SQuIRE^56^ and Telescope^57^, future works will need to assess its performance using 3’ end 10X data. Additionally, TEs can be integrated into genic transcripts (“TE-derived isoforms”) modulating their expression^3,58,59^. A misconception about 3’ end 10X data is that it cannot detect splicing events, however, STAR (one of the recommended aligners for SoloTE) has been recently shown to detect them^60^. Thus, by taking advantage of these capabilities provided by STAR, the locus-specific analysis of TEs done by our tool could further be improved to reveal TE-derived isoforms.

As proof of concept, we applied SoloTE to profile the TE transcriptome of the murine embryonic two-cell stage, during early GC, and in the APP/PS1 AD mouse model. In the case of the two-cell stage, where current evidence indicates activation of TEs, we were able to confirm known examples, along with ~80 other instances that, to the best of our knowledge, have not been reported previously. This highlights the capability of SoloTE in recovering the expected cellular distribution of TEs. Finally, for the last two biological conditions studies, we found that TE activity also seems to involve more clusters than just the disease-associated ones. Overall, this could suggest that, for such pathologies, TEs that become active might drive cell types towards such disease phenotypes. Locus-specific analysis revealed that TEs could be impacting several genes involved with the regulation of gene expression, and genes previously implicated with disease (as described for the early GC and the APP/PS1 AD results). Although these correlation analyses suggest potential mechanisms in which TEs could influence gene expression, they do not reveal a cause-effect link. Consequently, follow-up works with further experimental validation (i.e., gain- and/or loss-of-function experiments) are needed to confirm this hypothesis. Nonetheless, these analyses highlight the relevance of locus-specific analysis to predict the putative influence of TE activation on gene regulation.

Considering the ease of use of SoloTE, we expect it to be efficiently adopted and integrated into the current scRNA-Seq analysis pipelines used by the scientific community. This in turn will help to expand the understanding of how TE expression could impact cellular diversity.

## Methods

### SoloTE implementation

SoloTE takes as input files a BAM file resulting from the alignment against a reference genome, that has the GN and CB tags (i.e., resulting from the use of CellRanger or STAR^61^), and a TE annotation file in BED format. Two requirements for the BAM file are that unique and multi-mapping reads can be distinguished and that the multi-mapping reads must not be discarded. Accordingly, for the alignment process, we recommend using STAR with options --winAnchorMultimapNmax 100 (to increase sensitivity and detection of multi-mapped reads), --outFilterMultimapNmax 100 (to control whether multi-mapping reads are included in the output BAM file) and --outSAMmultNmax 1 (to report only the best alignment). We suggest these values based on a small experiment we performed (Supplementary Figure 5), where we found that setting them at 100 resulted in good results, with small improvements seen when using 500, in agreement with previous works^62–65^. Collectively, these alignment recommendations will allow for greater mapping of TE reads, and the distinction between unique and multi-mapping reads. Thus, for reads having multiple mapping locations, the best one is selected, and the remaining alignments are discarded.

The first step of SoloTE is the selection of reads not assigned to genes. Both CellRanger and STAR assign reads to known genes if the read is fully contained within one of its exons (or more than one exon if the alignment is spliced), and aligning on the same strand in which the gene is annotated. In turn, reads assigned to known genes are discarded, to avoid mistakenly reporting TEs fully contained within genes, as expressed. This behavior can be modified by using the “dual” option of SoloTE, in which reads assigned to genes will also be analyzed in the following steps. For each read that passed these filters, the overlap with the TE annotation is assessed using BEDtools^66^, and TE expression is summarized at the locus-level if the alignments have a MAPQ equal to or greater than a user-defined threshold (default = 255, only uniquely mapped reads), and then, at the family level if they do not meet the criteria (i.e., all multi-mapping reads per TE locus are aggregated into a single group corresponding to their family classification). The logic behind this idea is that most TEs have accumulated mutations that distinguish them from other instances^3,4^ (Fig. 1). After reads associated with TEs are annotated, a new matrix having both genes and TEs expression per cell is obtained as output, which is compatible with downstream analysis tools, such as Seurat^67^.

### Benchmarking and validation

To benchmark and validate SoloTE, we simulated data from the human genome hg38 version, and from the mouse genome mm10 version. For each genome, TEs were divided into young and old, if their divergence from the consensus sequence was ≤10% or >10%, respectively^56^. Then, for each TE group, a cell expression matrix of 1000 randomly selected TEs across 500 cells was generated using Minnow^68^. Additionally, Minnow generates FASTQ files that match the simulated expression matrix. These FASTQ files were then used as input to STAR, and the resulting BAM files were then processed. As these BAM files are also suitable for scTE, we were able to perform a comparison from the same set of alignments. scTE was run first in the “build” mode, to generate the indexes for the hg38 and mm10 genomes, with option “-mode inclusive”, and then, the main scTE pipeline was used with the resulting BAM files from the STAR alignment, with default options. Both SoloTE and scTE were run with 8 threads.

### Analysis of previously published datasets

The previously published datasets used in this work were the murine embryonic two-cell stage^9^, early GC^11^, and APP/PS1 AD mouse model^13^. The raw FASTQ files were obtained from the database in which the authors made them available (Gene Expression Omnibus for the first two, and AD Knowledge Portal for the last one, Supplementary Table 3). All alignments were performed using STAR v2.7.9a^61^. Mouse datasets were aligned to the mm39 genome, and human datasets to the hg38 genome. Cell demultiplexing was done using the Chromium 10X V2 whitelist for the embryonic 2-cell and early GC datasets, and using the Chromium 10X V3 whitelist for the APP/PS1 dataset. Afterwards, SoloTE was run in the resulting BAM files, using 8 processors. The gene and TE cell expression matrixes were first filtered, only keeping the cells selected in their respective original works, and these new matrixes were then processed with the Seurat v4.0.6 package^67^ of the R statistical computing environment^69^, using the default analysis pipeline. Finally, marker analysis was carried out using the “*FindAllMarkers*” function of Seurat, only keeping the results having adjusted *P*-value ≤0.05 and average log_2_(Fold Change) ≥1.

### Locus-specific analysis of marker TEs

Marker TEs identified unambiguously at specific genomic locations with SoloTE, were used for locus-specific analysis. TEs can potentially influence the activity of neighboring genes, and thus, to predict such impact we applied a computational strategy similar to previously published works^41,49,70^, which we describe next. First, we associated TEs with their closest gene using BEDtools^66^. Then, for each gene-TE pair, we applied a linear modeling strategy in which gene expression was the response variable and the TE expression was the explanatory variable, using the “lm” function of the R statistical computing environment. This allowed us to obtain which TEs could explain changes in gene expression in a statistically significant manner (model *P*-value ≤0.05). Then, for all of the significant gene-TE associations, the correlation between gene and TE expression was assessed using the “cor” function of R, to predict whether the TE could be impacting gene expression positively (i.e, positive correlation) or negatively (i.e, negative correlation). To understand the large-scale impact of these associations, the genes in statistically significant models were analyzed with the “Functional classification” tool of the Panther database^71^.

### Statistics and reproducibility

Statistical tests were applied during the “FindAllMarkers” step of Seurat in R statistical computing environment to find genes and TEs with higher expression in a cell cluster when compared to the other groups. The Wilcoxon Rank Sum test was used to test for statistical significance, followed by Bonferroni correction to obtain adjusted *P*-values. As indicated above, a threshold of adjusted *P*-value ≤0.05 was used to keep statistically significant results.

### Reporting summary

Further information on research design is available in the Nature Research Reporting Summary linked to this article.

## Supplementary information

Supplementary Material

Description of Additional Supplementary Files

Supplementary Data 1

Supplementary Data 2

Reporting Summary

## Data Availability

All sequencing datasets used in this study were obtained from public data repositories. The murine 2C-like data was obtained from the Gene Expression Omnibus (GEO) database, accession GSE114952. The early GC data was also obtained from GEO, accession GSE134520. The APP/PS1 AD mouse model was obtained from the AD Knowledge Portal, accession syn23763409. Detailed information, including accession URLs for these datasets, is available in Supplementary Table 3. All relevant data are available from the corresponding authors on reasonable request.

## Code availability

SoloTE is publicly available at https://github.com/bvaldebenitom/SoloTE/ and in Zenodo^72^.
