# Supplementary material for: SoloTE for improved analysis of transposable elements in single-cell RNA-Seq data using locus-specific expression
Source: Commun Biol. 2022 Oct 6;5:1063. doi: 10.1038/s42003-022-04020-5 (PMC9537157; doi:10.1038/s42003-022-04020-5)
Supplement: Supplementary file 1 — Supplementary Material [file 42003_2022_4020_MOESM1_ESM.pdf]

SoloTE for improved analysis of Transposable Elements in single cell RNA-Seq data using locus-specific expression

Rocío Rodríguez-Quiroz[1], Braulio Valdebenito-Maturana[1,*]

[1] Department of Genetics and Genomic Sciences, Icahn School of Medicine at Mount Sinai, New York, New York, USA.
*Corresponding author. Email: braulio.valdebenitomaturana@mssm.edu

Contents of this file

Supplementary Figure 1
Supplementary Figure 2
Supplementary Figure 3
Supplementary Figure 4
Supplementary Figure 5
Supplementary Table 1
Supplementary Table 2
Supplementary Table 3

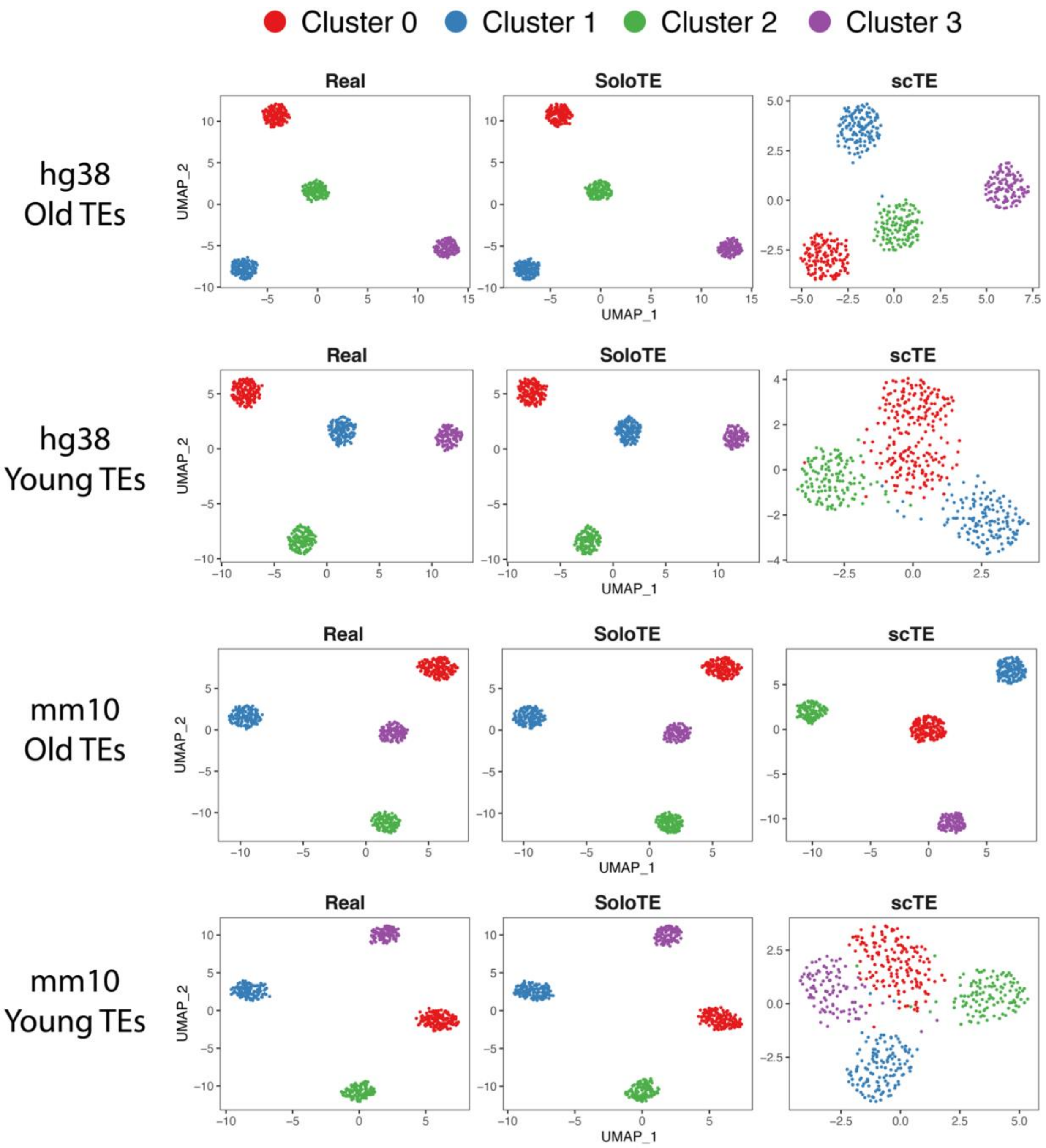

Supplementary Figure 1. UMAP dimensional reduction plots for each of the simulated datasets using the Seurat Integration pipeline. The data from which the UMAP plots were generated is indicated above each one of them. Clusters are colored in red (Cluster 0), blue (Cluster 1), green (Cluster 2), purple (Cluster 3).

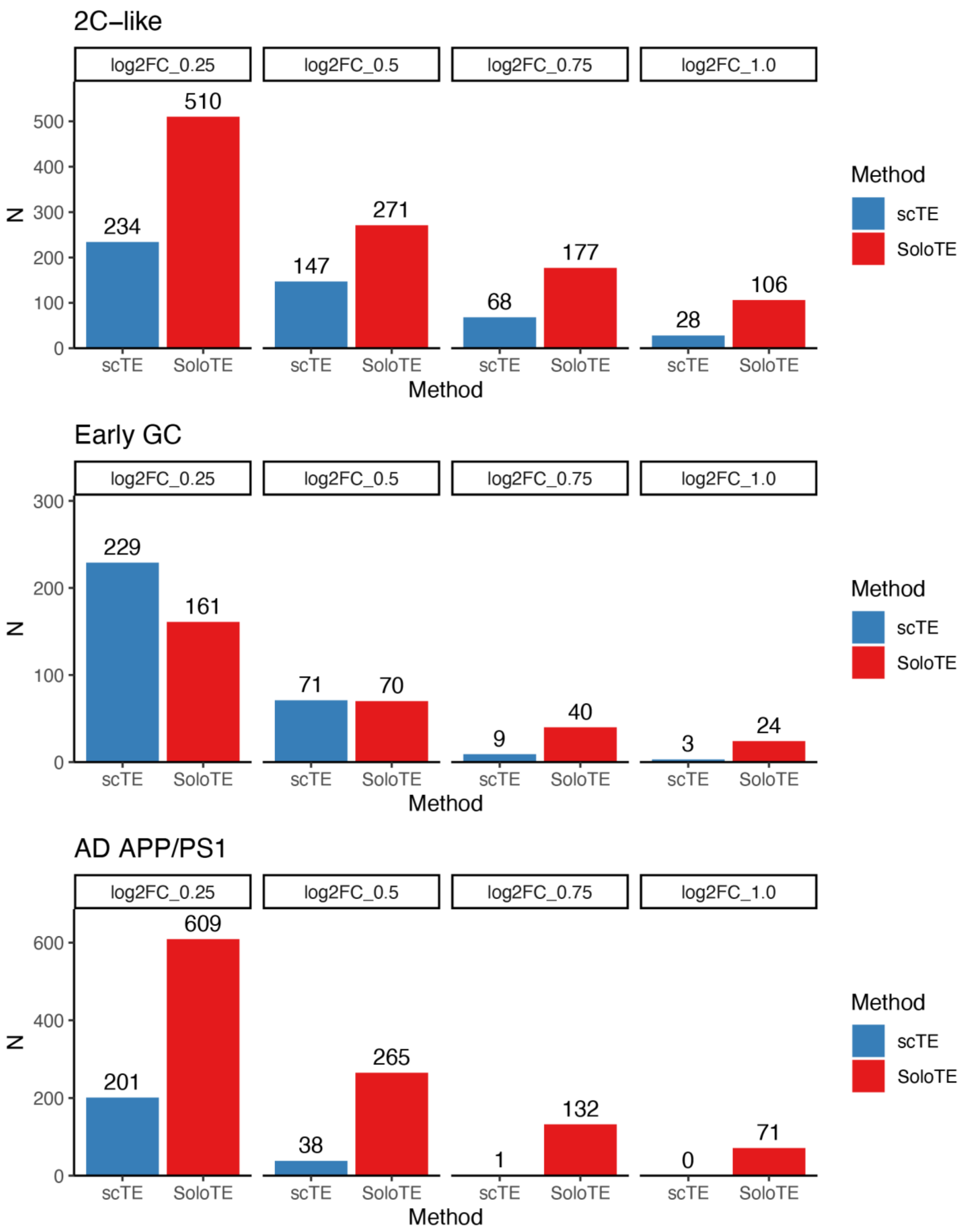

Supplementary Figure 2. Number of marker TEs detected when using scTE (blue bars) or SoloTE (red bars) at different log_2(Fold Change) thresholds: 0.25, 0.5, 0.75 and 1.0. Each panel corresponds to the experimental dataset analyzed: 2C-like, Early gastric cancer (Early GC), and Alzheimer's Disease APP/PS1 mice.

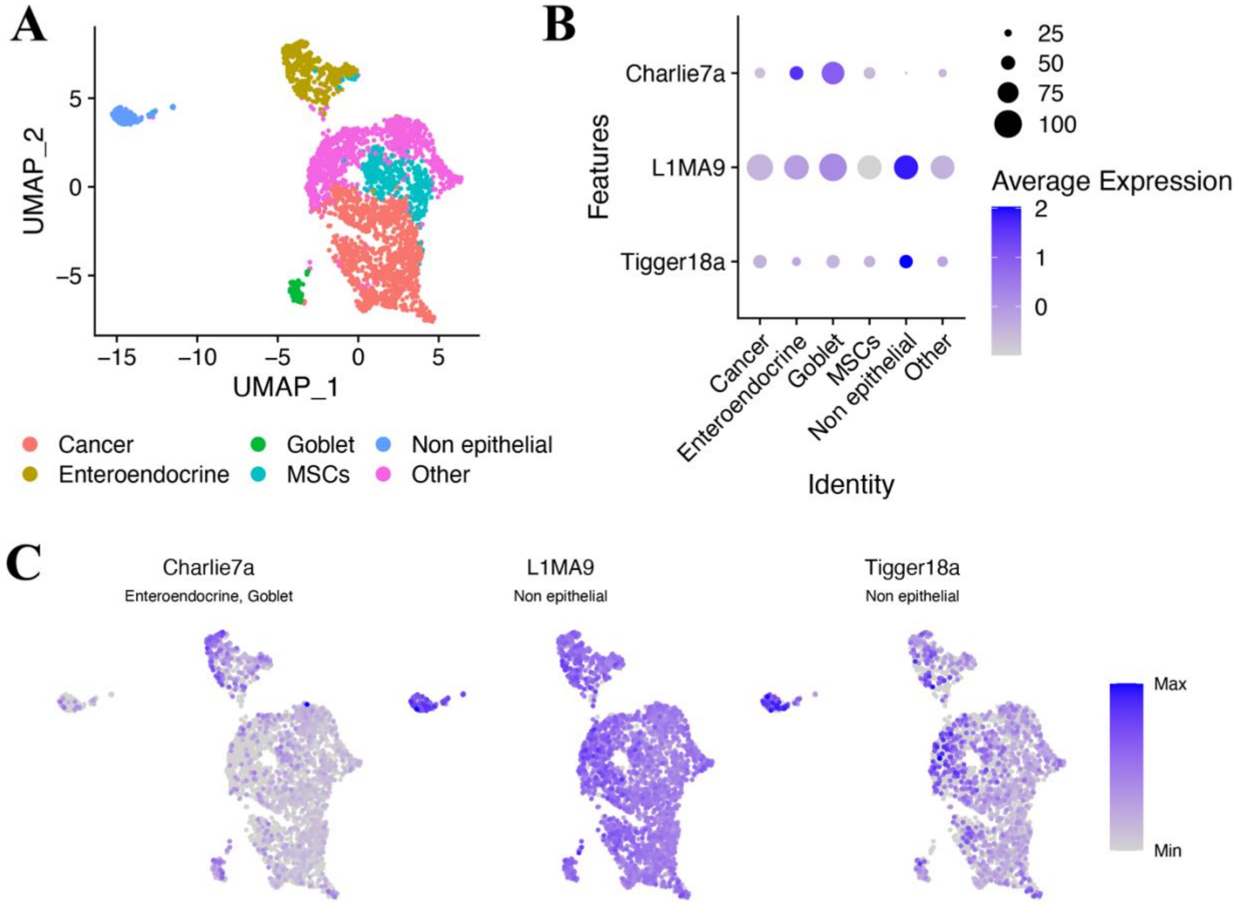

Supplementary Figure 3, TE expression detected with scTE during early gastric cancer. A. UMAP plot indicating the different cell types. **B.** Dot plot depicting marker TEs per cell types (indicated on the x-axis). **C.** UMAP plots of marker TEs. Label below the TE identifier indicates the cluster at which the TE is a marker.

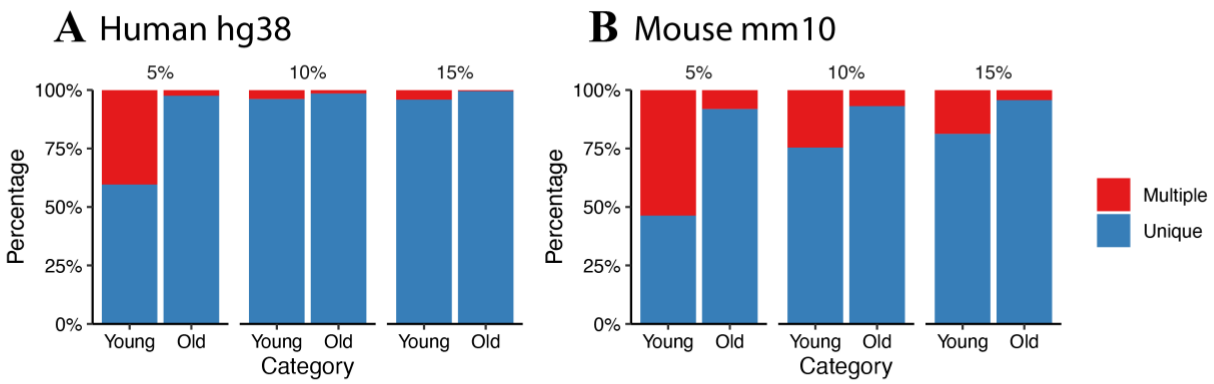

Supplementary Figure 4. Percentage of Unique (blue) and Multiple (red) mapped reads, using different thresholds (5%, 10% and 15%) to label TEs "Young" or "Old". **A.** Results in the Human hg38 genome version. **B.** Results in the Mouse mm10 genome version.

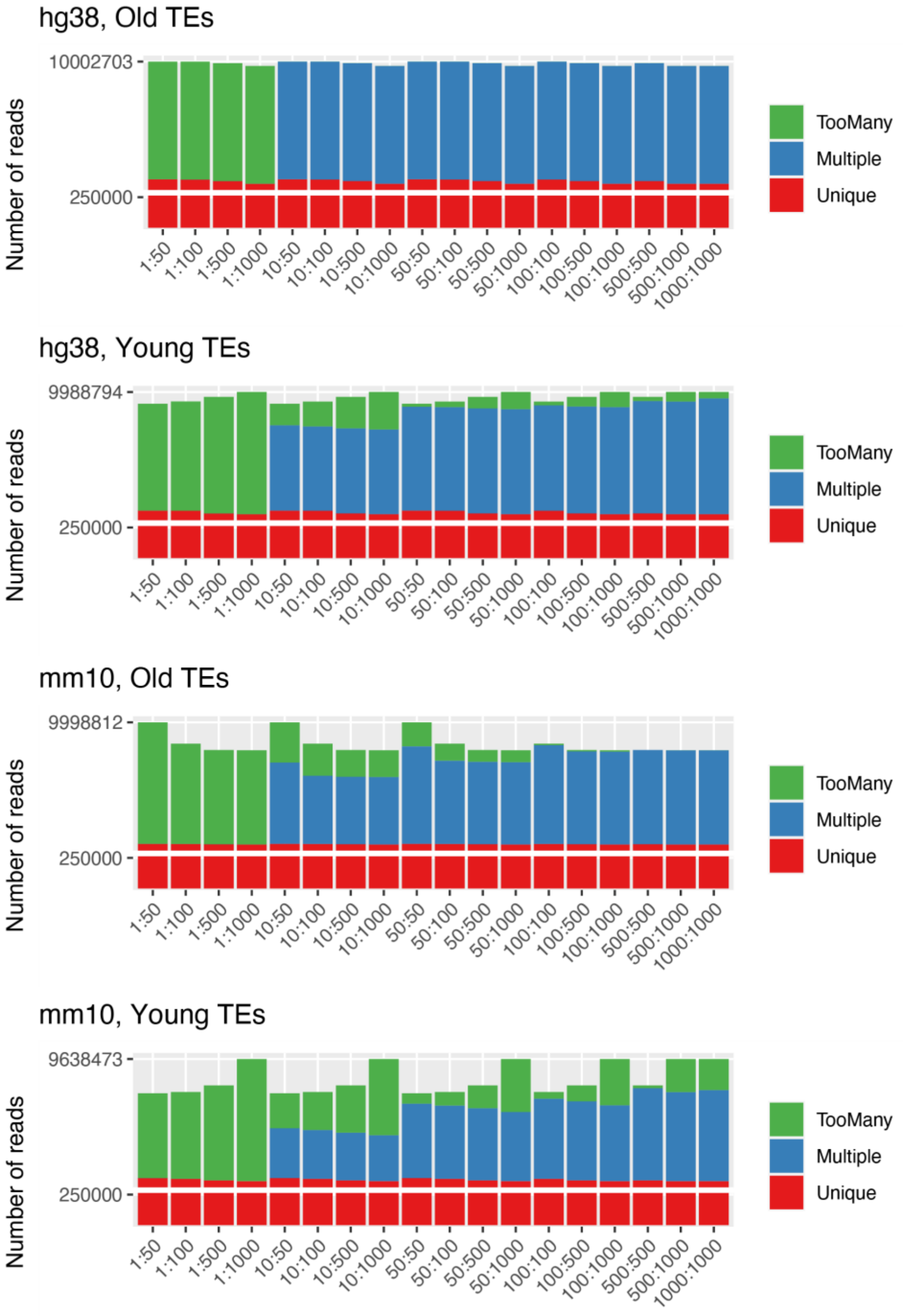

Supplementary Figure 5. Assessment of the reads reported in each category by STAR, when varying the parameters “outFilterMultimapNmax”and “winAnchorMultimapNmax”. “Unique” (red): read aligning uniquely; “Multiple” (blue): read aligning in multiple locations and reported in the BAM file; “TooMany” (green); read aligning in multiple locations, but not reported in the BAM file because the number of locations exceed the value of “outFilterMultimapNmax”. We performed the experiment aligning our simulated data against their respective reference genome. On the x-axis the combination of the “outFilterMultimapNmax”and “winAnchorMultimapNmax” is depicted, separated by “:” (for example, 1:50 indicates that “outFilterMultimapNmax” is set at 1, and “winAnchorMultimapNmax” is set at 50).

Supplementary Table 1. scTE read assignment across its different modalities.

**Identifier**	**Exclusive**	**Inclusive**	**No_intron**	**Type**
LTR48B	0	3450	0	TE
HIST1H4I	115	115	115	Gene
AluSz6	11	11	0	TE
HIST1H2AG	4	4	4	Gene
AluY	5	6	0	TE
AluSg	4	4	0	TE
AluSq	3	3	0	TE
AluJr	2	2	0	TE
FAM	2	2	0	TE
L1PA3	2	2	0	TE
ABT1	1	1	1	Gene
LINC00240	1	1	1	Gene
MLT1H	0	2	0	TE
AluSc8	1	1	0	TE
AluSp	1	1	0	TE
AluSx	1	1	0	TE
AluSx1	1	1	0	TE
AluSz	1	1	0	TE
LTR6B	1	1	0	TE
MLT1A	1	1	0	TE
MIR	0	1	0	TE

Resulting counts per gene or TE when using scTE with a small BAM file. “Identifier” corresponds to the identifier of the gene or TE (indicated in the “Type” column). The 3 modalities of scTE are depicted: “Exclusive”, “Inclusive”, “No_intron”.

Supplementary Table 2. scTE index building running time.

**Index**	**Time (min.)**
hg38 exclusive	5
hg38 inclusive	8
hg38 no intron	42
mm10 exclusive	5
mm10 inclusive	8
mm10 no intron	7

The first column describes the genome and index modality, and the second the time it took to build the index required by scTE.

Supplementary Table 3. Accession URL of the experimental datasets used in our work.

**Dataset**	**Publication DOI**	**Accession URL**
Murine embryonic 2-cell stage	10.1016/j.stem.2018.05.025	https://www.ncbi.nlm.nih.gov/geo/query/acc.cgi?acc=GSE114952
Early gastric cancer	10.1016/j.celrep.2019.04.052	https://www.ncbi.nlm.nih.gov/geo/query/acc.cgi?acc=GSE134520
APP/PS1 Alzheimer's disease mouse model	10.1016/j.celrep.2021.108739	https://doi.org/10.7303/syn23763409
