# Supplementary material for: SoloTE for improved analysis of transposable elements in single-cell RNA-Seq data using locus-specific expression
Source: Commun Biol. 2022 Oct 6;5:1063. doi: 10.1038/s42003-022-04020-5 (PMC9537157; doi:10.1038/s42003-022-04020-5)
Supplement: Supplementary file 2 — Description of Additional Supplementary Files [file 42003_2022_4020_MOESM2_ESM.pdf]

Description of Additional Supplementary Files

File name: Supplementary Data 1

Description: Marker analysis for each of the biological conditions studied, using the TE expression obtained with SoloTE or scTE.

File name: Supplementary Data 2

Description: Analysis of genes statistically associated with SoloTE locus-specific TEs, across all the biological conditions studied.
